# Validation of the Instagram Addiction Scale in Greek Youth

**DOI:** 10.14806/ej.26.1.973

**Published:** 2021-08-23

**Authors:** Maria Zarenti, Flora Bacopoulou, Maria Michou, Ioulia Kokka, Dimitrios Vlachakis, George P. Chrousos, Christina Darviri

**Affiliations:** 1 Postgraduate Course of Science of Stress and Health Promotion, School of Medicine, National and Kapodistrian University of Athens, Athens, Greece; 2 University Research Institute of Maternal and Child Health & Precision Medicine and UNESCO Chair on Adolescent Health Care, National and Kapodistrian University of Athens, Aghia Sophia Children’s Hospital, Athens, Greece; 3 Laboratory of Genetics, Department of Biotechnology, School of Applied Biology and Biotechnology, Agricultural University of Athens, Athens, Greece; 4 Lab of Molecular Endocrinology, Center of Clinical, Experimental Surgery and Translational Research, Biomedical Research Foundation of the Academy of Athens, Athens, Greece

## Abstract

Instagram is one of the fastest growing social networking platforms. A body of evidence suggests that Instagram problematic use and addiction have negative effects on the psychological well-being of young people. The Instagram Addiction Scale, a self-report tool assessing Instagram problematic use and addiction, has been developed recently. The aim of the present study was to validate the Instagram Addiction Scale in the Greek language and to assess its psychometric properties. An online and on-print self-report survey was conducted among Greek youth, aged between 18 and 24 years. The survey included the Instagram Addiction Scale, the Rosenberg Self-esteem Scale, the Perceived Stress Scale, and the Big Five Inventory. A total of 967 respondents participated in the study. The analysis suggested that the Greek version of the Instagram Addiction Scale has good psychometric properties. The principal component factor analysis for construct validity generated two subscales as the original instrument: social effect and impulsion. Internal consistency for the two subscales and the scale’s total score was satisfactory, with Cronbach’s α at 0.76, 0.85 and 0.88, respectively. Correlation analyses revealed positive associations between the perceived stress scale and social effect, and the Instagram Addiction Scale’s total score (p<0.0001 and p=0.002 respectively). This is the first study validating the Instagram Addiction Scale in Greek youth, which can be used by researchers and practitioners for the evaluation of youth problematic use of Instagram.

## Introduction

Over the last two decades, the use of Social Networking Sites, such as Facebook, Twitter and Instagram, has evoked rapidly. Research has shown that social media use may improve human interaction, psychological well-being and the learning process (Baumer, 2013; Garrett and Cutting, 2012; Baumöl et al., 2016; Schultz, 2016; Hutter et al., 2013). Nevertheless, a growing body of evidence has shown that a negative side of social media also exists (Mäntymäki, and Islam, 2016). The adverse effects of social media pertain to several domains *i.e.*, satisfaction with life (Satici, 2019; Satici and Uysal, 2015), loneliness (Błachnio et al., 2016; Ryan and Xenos, 2011), academic performance (Al-Yafi et al., 2018; Junco and Cotten, 2012), and low self-esteem (Hawi and Samaha, 2017).

Specifically, Instagram, with more than a billion active users worldwide and more than 500 million daily users, represents one of the fastest growing social networking platforms and the most popular among young people with more that 59% of its users being between 18 and 29 years old (Alhabash and Ma, 2017). Casaló *et al.* attributed the success and popularity of Instagram among young adults to the fact that the platform enables users to gain instant popularity and co-create value with opinion leaders (Casaló et al., 2018).

According to research conducted by the Royal Society for Public Health and the UK’s Youth Health Movement, Instagram is considered the most negatively affecting social media platform in terms of its impact on young people’s mental health. Specifically, both positive and negative effects of all social media on the health of young people were investigated. The study involved 1,479 young people aged 14 to 24 years. Participants rated popular applications on topics related to stress, depression, loneliness, bullying and body image. According to the results of the research, the YouTube platform emerged as the most positive, whereas Instagram and Snapchat were considered particularly harmful to the mental health and well-being of young people. However, a good aspect of these two applications has been reported by Cramer; Instagram was found to have a positive effect on self-expression and self-identity. Based on the findings of the Royal Society for Public Health and the UK Youth Health Movement, public health experts called social media to introduce a series of tests and measures, such as pop-up windows, to warn users when using social media for a long time and to protect mental health (supported by 70% of young people surveyed) (Cramer, 2017). Another suggestion addressed to the social media platforms was to develop user tracking tools for mental health issues, and thus urge users to seek help from mental health professionals. Similarly, other studies (Zalsman et al., 2016; Whitaker et al., 2017) concluded that the use of social media can help in the early diagnosis of depression. De Choudhury *et al.*, analysed the profile of 476 people on Twitter and created a protocol that could predict depression before serious symptoms appeared in 7 out of 10 cases (De Choudhury et al., 2013).

Contrary to this preventive to mental health use of Instagram, a growing body of evidence indicates some concerning effects from the excessive use of this platform. Although social media platform use is a rising phenomenon during the last decade, a psychiatric diagnosis for “internet addiction” or “social media addiction” does not yet exist. Nonetheless, even informally, the term “addiction” is used and investigated thoroughly in terms of social media use among teenagers and young adults.

One of the reasons why “internet addiction” has not yet come to light as a formal term is because it is not a substance. However, in terms of behavioural addiction the outcomes are similar to the ones caused by a substance (Griffiths, 2005). In excessive “doses’, negative effects may arise and become addictive, especially in adolescents and young adults (Griffiths et al., 2016; World Health Organization, 2011). Most frequently observed symptoms which resemble those of substance abuse include compulsive behavioural involvement, lack of motivation to engage in other activities, and mental and physical symptoms when “deprivation” from the platforms is attempted (D’Arienzo et al., 2019). These symptoms are common among undergraduate students who are shy and prefer the online to the offline world (Orr et al., 2009). This “virtual reality” is also very tempting for individuals with social anxiety (Buote et al., 2009) or depression (Andreassen et al., 2016).

Despite Instagram’s addictive aspects and negative effects on the physical and mental well-being, only recently a self-report questionnaire aiming to assess Instagram problematic use and addiction was developed. Kircaburun and Griffiths developed the Instagram Addiction Scale (IAS) by using a modified version of the Internet Addiction Test (Young et al., 1999) and found that IAS had a satisfying internal consistency when evaluated in a sample of 752 university students. Furthermore, Instagram addiction was negatively associated with agreeableness, conscientiousness, and self-liking, whereas daily Internet use was positively associated with Instagram addiction (Kircaburun and Griffiths, 2018).

## Materials, Methodologies and Techniques

### Translation procedure

The first step of the validation process was to receive authorization by Kircaburun K. and Griffiths M.D. The translation procedure was then performed according to the World Health Organization’s guidelines (WHO, 2020) by an expert panel. When all the forward-backwards steps were completed by the panel, a test-pretest of the questionnaire was conducted to identify unclear expressions. Participants in this test (20 males, 20 females) were representative of the study’s population, with regards to age and native language. The Greek version of the instrument was then finalised.

### Participants and procedures

This study was performed in the province of Attica, Greece in November of 2020. Participant inclusion criteria were age between 18 and 24 years, and ability to read and write in the Greek language. The questionnaire was distributed mainly online on Google Forms. Online distribution was held through various social media platforms, mostly via Facebook, whereas a printed form of the questionnaire was distributed to various universities.

### Ethical considerations

The study’s protocol was approved by the ethics committee of the Medical School of the National and Kapodistrian University of Athens and was in accordance with the 1975 Helsinki Declaration. For the online version, a brief text of the study’s protocol informed the respondents about the study’s aim and submission of their response was considered as an online consent. For the printed version, respondents were informed and signed a consent form prior to participation.

### Measures

#### Sociodemographic characteristics:

Participants were asked about their sex, age, family and income status as well as their educational level.

#### Instagram Addiction Scale (IAS):

IAS consists of 15 items and provides a distinguishing cut off point for Instagram addictive and non-addictive users. Answers are given on a 6-point Likert scale ranging from “never” to “always” and score can range from 15 to 90. The cut-off points were determined as following: scores between 15-37 classify participants as non-addicts, scores ranging from 38 to 58 as mildly addicted, moderately addicted are those who score from 59 to 73, and scores above 73 indicate severe addiction (Kircaburun and Griffiths, 2018).

#### Perceived Stress Scale-14 (PSS):

This is a 14-item questionnaire that measures the self-reported level of stress. Answers are given on a 5-point Likert scale, ranging from 0=never to 4=very often. Seven items are considered positive and the other seven negative. Total scores are calculated after reversing the scores for the positive items and then summing all answers’ scores. Higher scores indicate higher levels of stress. The PSS has been translated and validated in the Greek language with good psychometric properties and a satisfying Cronbach’s α coefficient (0.82) (Andreou et al., 2011).

#### Big Five Personality Inventory (BFI):

This is a 5-point Likert scale that includes 44 items, allowing the assessment of the five personality dimensions. Participants rate each BFI item on a 5-point scale ranging from 1 (disagree strongly) to 5 (agree strongly); scale scores are computed as the participant’s mean item response. The model of 44 questions measuring the personality traits, has been translated in the Greek language and used in the present study (Panayiotou et al., 2004).

#### Rosenberg Self-Esteem Scale:

This is a 4-Likert scale including 10 items. The scale measures self-worth by examining both positive and negative feelings about the respondent’s self. Scores between 15 and 25 are considered average. The Greek version of the Rosenberg Self-Esteem Scale was used in this research (Galanou et al., 2014).

### Data analysis

Data is presented as N (%) for qualitative variables, and as mean (SD) for quantitative variables. Principal component analysis (PCA) was conducted to extract the factors of the IAS scale. Sample’s adequacy and the correlation among the items were tested with the Kaiser-Meyer-Olkin measure and Bartlett’s test of sphericity, respectively. The varimax rotation method was used and when questions’ loadings were greater than 0.3, the items were assigned into specific factors. Cronbach’s alpha was calculated to examine internal consistency. Independent samples t-test and ANOVA test were conducted to evaluate differences between groups. Correlations between IAS subscales, as well as between IAS subscales and other measurements of the study were calculated. Pearson’s rho coefficient was used to assess correlations between quantitative variables. SPSS v.24 for Windows was used to perform statistical analyses and the level of significance for all analyses was 0.05.

## Results

A total of 967 valid responses were collected. Participants’ sociodemographic characteristics and descriptive statistics for the Big-Five Personality Inventory, Rosenberg’s Self-Esteem Scale and Perceived Stress Scale scores are presented in Table 1. In total, 156 participants were between 18 and 20 years old, 297 participants were between 20 and 22 years old and 514 participants were between 22 and 24 years old. The majority of participants were females (87.1%), unmarried (98.1%), had a Bachelor’s degree (64.1%) and cohabitated (67.4%).

The results of the Principal Component Analysis (PCA) of the 14 items with orthogonal rotation (varimax) are presented in Table 2. It was observed that the 14 items were able to explain 48.45% of the total variance and the scale was composed by two factors with eigenvalue greater than Kaiser’s criterion of 1. The two factors were named as social effect and compulsion consisting of seven (minus one question comparing to the original subscale) and seven items, respectively. The sampling adequacy for the analysis was verified with the Kaiser-Meyer-Olkin measure (KMO = 0.914). Furthermore, Bartlett’s test of sphericity, x^2^(105) = 5382,20, p<0.0001, showed that correlations between items were sufficiently large to perform PCA. The item “How often do you form new relationships with fellow Instagram users?” did not load adequately to any of the factors and it was excluded from the final list of items. Cronbach’ α coefficients for social effect, compulsion and total scale were 0.76 and 0.85, respectively, indicating satisfactory internal consistency.

Table 3 presents descriptive statistics for the two IAS subscales and the total IAS score. Correlations between the social effect and compulsion subscale and total IAS scores are presented in Table 4. Overall, a strong positive correlation between social effect and compulsion was found, indicating that negative effects from the Instagram addictive use on individuals’ real-life relationships are associated to their increasing need to use the platform. The Spearman’s correlation coefficient between social effect and compulsion, social effect and total scale, and compulsion and total scale were 0.66, 0.88 and 0.94, respectively.

Table 5 presents associations between social effect, compulsion, IAS total score and the study variables. Overall, there were statistically significant differences between males and females, with females scoring higher in all scales. Age groups and educational level subscales did not seem to present any significant differences. Statistically significant correlations were found between perceived stress and social effect (p<0.0001) and total IAS score (p=0.002). From the BFI scale, pleasantness score was negatively associated to social effect and total IAS score (p<0.0001 and 0.030 respectively), and conscientiousness score was also negatively associated to social effect, impulsion, and total IAS scores (p<0.0001 for all). Finally, neuroticism score was positively associated to social effect, impulsion, and total IAS scores (p<0.0001, p=0.011 and p<0.0001 respectively).

## Discussion

The development of the IAS addressed the need of measuring individuals’ addiction to Instagram, one of the most widely used social networking sites, in view of emerging evidence supporting that problematic Instagram use can lead to addiction. The aim of the current study was to validate the Instagram Addiction Scale in a Greek youth population sample, aged between 18 and 24 years, and evaluate its psychometric properties.

In line with the findings of Kircaburum and Griffiths (2018), the PCA resulted in two factors: 1. Social effect, consisting of eight items, and 2. Compulsion, consisting of seven items. The social effect sub-factor reflected negative effects from the Instagram use on individuals’ real life social relations and on networking (*i.e.*, “How often do you prefer the excitement of Instagram instead of being with your close friends?”). The compulsion sub-factor reflected the increased need for Instagram use, the frequency of forgetting about time while logged on to Instagram, and the avoidance of real-life concerns using Instagram (*i.e.*, “How often do you try to cut down the amount of time you spend on Instagram and fail?”). Both factors showed satisfactory internal consistency. Strong correlation was found between social effect and compulsion indicating that they collectively and cooperatively represented individuals’ problematic use and addiction to Instagram. Correlation analyses between IAS subscales and IAS total score, and participants’ sociodemographic characteristics revealed differences between males and females, with greater scores in females in both subscales as well as in the IAS total score.

Positive strong correlations were found between perceived stress and the IAS subscale regarding negative effect, as well as between perceived stress and total IAS score. This finding supports previous research showing that social networking sites constitute a source of stress (Maier et al., 2012). With respect to Instagram, it has been recently shown that time spent on Instagram is a significant predictor of stress (Lowe-Calverley et al., 2019). Similarly, Sanz-Blas *et al.*, (Sanz-Blas et al., 2019) in a study of 342 active Instagram users examined the negative impact of excessive use of Instagram on individuals’ psychological well-being and found that Instagram overuse resulted in elevated levels of stress and emotional fatigue. A question, therefore, emerges regarding the mechanisms that underlie the association between stress and use of Instagram. As new technologies and incoming information increase, individuals feel unable to absorb all the amount of information and thus experience more stress (Wurman, 1989). The loss of information that derives from the gap between the available information and the users’ cognitive capacity may lead to discomfort, negative feelings, and increased activation (Ragu-Nathan et al., 2008). With regards to Instagram, as new information is constantly updated on the platform, heavy Instagram users may experience incompetence and thus overuse the platform to access more information (Hong et al., 2014). Furthermore, for individuals with high perceived stress who tend to perceive life events as stressful, Instagram with its colorful photos and videos from all over the world portrays a safe escape from real life.

Neuroticism, the individual temperamental tendency towards anxiety, self-doubt, and depression, is closely related to the psychological construct of stress. The present study revealed significant associations between participants’ perceived stress and level of neuroticism, and addiction to Instagram. According to Ershad and Aghajani (Ershad and Aghajani, 2017), elevated levels of individuals’ neuroticism increase the probability of Instagram social networking. These findings are in line with the results of Wang *et al.* (Wang et al., 2015), who found that higher neuroticism is associated with internet addiction in general. It was hypothesized that since neurotic individuals are interested in what other people think or say about them, they tend to spend too much time on Instagram by stalking others’ profiles or reading comments, which may result in addiction (Choi et al., 2017).

Furthermore, non-significant results were found regarding the extraversion subscale of the Big Five Inventory Scale and Instagram Addiction Scale. This finding verified the study of Wang *et al.* (Wang et al., 2015) who found an opposite-direction relationship between extraversion and addictive use of social media. Yang (Yang, 2016) found that higher loneliness, which is related to lower extraversion (Cheng and Furnham, 2002), was associated to increased photograph and video sharing on Instagram. In the study of Kircaburum and Griffiths (2018), participants’ extraversion was not related to IAS scores. With regards to this finding, it is hypothesized that extraverted individuals could reveal a tendency towards Instagram addiction, as they could become dependent on the popularity and excessive chances for interaction that a platform like Instagram can offer.

Finally, regarding the dimension of self-esteem, no strong correlations with the IAS subscales were found. This outcome was surprising, because it is common for people with low self-esteem to spend an increased amount of time on social media. A possible explanation is that self-image and self-esteem may improve through posting (Błachnio et al., 2016).

This study has some limitations. Confirmatory Factor Analysis was not performed, which could have validated the study’s findings further. Also, a test-retest design was not included to assess variability between measurements. Although a large sample was employed, generalization of the results cannot be easily verified because the sample was recruited mainly from the capital of Athens and was not representative of the entire youth population of Greece.

In conclusion, IAS is a newly introduced instrument aiming to evaluate problematic use and addiction to Instagram that is considered to adversely affect young people’s mental health. This is the first study validating the Instagram Addiction Scale in Greek youth, which can be used by researchers and practitioners for the evaluation of youth problematic use of Instagram. Future research studies are needed to compare IAS to other standardised tests measuring youth addictive behaviours and personality traits, and include youth samples deriving from different socioeconomic and cultural backgrounds.

## Figures and Tables

**Table 1.** Participants’ sociodemographic characteristics and descriptive statistics for Big-Five Personality Inventory, Rosenberg’s Self-Esteem Scale and Perceived Stress Scale (PSS) scores.

Sex	N (%)	Big-Five Personality Inventory Score	Mean (SD)
Females	842 (87.1)		
Males	125 (12.9)		
		
**Age Groups**		Extroversion	3.13 (0.63)
18-20 years	156 (16.1)	Pleasantness	3.78 (0.51)
20-22 years	297 (30.7)	Conscientiousness	3.56 (0.60)
22-24 years	514 (53.2)	Neuroticism	3.38 (0.68)
		Openness	3.20 (0.54)

**Marital status**		**Rosenberg’s Self-Esteem Scale Score**	27.00 (7.00)
Unmarried	949 (98.1)		
Married	15 (1.6)	PSS Score	31.00 (8.90)
Divorced	3(0.3)		
		
**Education level**			
High School	4 (0.4)		
Lyceum	97 (10.0)		
IVET/IPS	143 (14.8)		
BSc	620 (64.1)		
MSc	103 (10.7)		
		
**Cohabitation**			
Yes	652 (67.4)		
No	315 (32.6)		

**Table 2.** Rotated factor loadings of the principal components analysis (PCA) for 14 Instagram Addiction Scale items (N=967).

	Social Effect Subscale	Compulsion Subscale
**1. How often do you prefer the excitement of Instagram instead of being with your close friends?**	0.703	
**2. How often do you form new relationships with fellow Instagram users?**	-	-
**3. How often do you become defensive or secretive when anyone asks you what you do on Instagram?**	0.409	
**4. How often do your grades or schoolwork suffer because of the amount of time you spend on Instagram?**	0.415	
**5. How often do you snap, yell, or act annoyed if someone bothers you while you are on Instagram?**	0.668	
**6. How often do you try to hide how long you have been on Instagram?**	0.606	
**7. How often do you choose to spend more time on Instagram over going out with others?**	0.759	
**8. How often do you feel depressed, moody or nervous when you are not on Instagram, which goes away once you are back on Instagram?**	0.574	
**9. How often do you try to cut down the amount of time you spend on Instagram and fail?**		0.637
**10. How often do you check your Instagram before something else that you need to do?**		0.778
**11. How often do you block out disturbing thoughts about your life with soothing thoughts of the Instagram?**		0.516
**12. How often do you find yourself anticipating when you will go on Instagram again?**		0.633
**13. How often do you fear that life without the Instagram would be boring, empty, and joyless?**		0.528
**14. How often do you lose sleep due to late night log-ins to Instagram?**		0.782
**15. How often do you find yourself saying “just a few more minutes” when on Instagram?**		0.786
**Eigenvalues**	5.952	1.316
**% of Variance**	39.679	8.775
**Croncbach’s α**	0.761	0.853

**Table 3.** Descriptive characteristics of the two subscales of IAS and total IAS score.

Subscale	Items	Range	Mean	SD	Minimum	Maximum
**Social Effect score**	7	6-42	12.25	5.17	7	39
**Compulsion score**	7	6-42	15.43	6.97	7	40
**Total IAS score**	14	6-84	27.68	11.08	14	78

IAS: Instagram Addiction Scale

**Table 4.** Correlations (Spearman’s rho) between IAS subscales and total IAS score.

	Social Effects core	Compulsions core	Total IAS score
**Social Effects core**	1		
**Compulsion score**	0.660**	1	
**Total IAS score**	0.882**	0.937**	1

IAS: Instagram Addiction Scale

**Table 5.** Associations between IAS subscales and total score and other study variables.

Study measurements	Categories	Social Effect score	Compulsion score	Total IAS score
**Sex**	Males	11.74 (4.99)	13.51 (6.00)	25.25 (9.94)
	Females	12.33 (5.19)	15.71 (7.06)	28.04 (11.20)
	p-value	0.232	<0.0001	0.004
**Age groups**	18-20 years	13.22 (5.30)*	16.71 (7.45)*	29.94 (11.59)*
	20-22 years	12.54(5.33)	15.53(6.78)	28.07(11.11)
	22-24 years	11.79 (4.99)*	14.98 (6.88)*	26.77 (10.81)*
	p-value	0.005	0.023	0.006
**Educational level**	High School	12.25 (2.50)	12.25 (4.65)	24.50 (7.00)
	Lyceum	12.06 (4.84)	14.52 (7.10)	26.58(11.18)
	IVET/IPS	11.47 (4.82)	14.34 (6.53)	25.80 (10.23)
	BSc	12.39 (5.10)	15.79 (6.95)	28.18 (10.92)
	MSc	12.70 (6.31)	15.76 (7.40)	28.46 (12.87)
	p-value	0.254	0.095	0.129
**Marital status**	Unmarried	12.24 (5.19)	15.44 (6.98)	27.69 (11.14)
	Married	12.07 (3.77)	15.20 (6.06)	27.27 (7.25)
	Divorced	15.67 (3.21)	11.67 (6.43)	27.33 (9.07)
	p-value	0.515	0.640	0.988
**PSS Total**	Spearman rho	0.138	0.057	0.100
	p-value	<0.0001	0.076	0.002
**Self-Esteem Total**	Spearman rho	−0.51	−0.001	−0.24
	p-value	0.113	0.981	0.451
**Extroversion Score**				
	Spearman rho	−0.44	0.031	−0.01
	p-value	0.169	0.332	0.975
**Pleasantness Score**	Spearman rho	−0.129	−0.015	−0.70
	p-value	<0.0001	0.636	0.030
**Conscientiousness Score**	Spearman rho	−0.171	−0.117	−0.153
	p-value	<0.0001	<0.0001	<0.0001
**Neuroticism Score**	Spearman rho	0.134	0.081	0.114
	p-value	<0.0001	0.011	<0.0001
**Openness Score**	Spearman rho	−0.041	0.013	−0.011
	p-value	0.201	0.675	0.739

IAS: Instagram Addiction Scale; PSS: Perceived Stress Scale-14

## Key Points

- Instagram is one of the fastest growing social networking platforms.
- Instagram Addiction Scale (IAS) is a self-report tool assessing Instagram problematic use and addiction.
- The Greek version of the IAS demonstrated satisfying internal consistency.
- The Greek version of IAS can be used for the evaluation of youth problematic use of Instagram.
